# ROS in Platelet Biology: Functional Aspects and Methodological Insights

**DOI:** 10.3390/ijms21144866

**Published:** 2020-07-09

**Authors:** Elena Masselli, Giulia Pozzi, Mauro Vaccarezza, Prisco Mirandola, Daniela Galli, Marco Vitale, Cecilia Carubbi, Giuliana Gobbi

**Affiliations:** 1 Department of Medicine and Surgery, University of Parma, 43126 Parma, Italy; elena.masselli@unipr.it (E.M.); giulia.pozzi@unipr.it (G.P.); prisco.mirandola@unipr.it (P.M.); daniela.galli@unipr.it (D.G.); giuliana.gobbi@unipr.it (G.G.); 2 School of Pharmacy and Biomedical Sciences, Faculty of Health Sciences, Curtin University, Bentley, Perth, WA 6102, Australia; mauro.vaccarezza@curtin.edu.au

**Keywords:** platelet, ROS, mitochondria, oxidative stress

## Abstract

Reactive oxygen species (ROS) and mitochondria play a pivotal role in regulating platelet functions. Platelet activation determines a drastic change in redox balance and in platelet metabolism. Indeed, several signaling pathways have been demonstrated to induce ROS production by NAPDH oxidase (NOX) and mitochondria, upon platelet activation. Platelet-derived ROS, in turn, boost further ROS production and consequent platelet activation, adhesion and recruitment in an auto-amplifying loop. This vicious circle results in a platelet procoagulant phenotype and apoptosis, both accounting for the high thrombotic risk in oxidative stress-related diseases. This review sought to elucidate molecular mechanisms underlying ROS production upon platelet activation and the effects of an altered redox balance on platelet function, focusing on the main advances that have been made in platelet redox biology. Furthermore, given the increasing interest in this field, we also describe the up-to-date methods for detecting platelets, ROS and the platelet bioenergetic profile, which have been proposed as potential disease biomarkers.

## 1. Introduction

Reactive oxygen species (ROS) and reactive nitrogen species (RNS) are highly reactive molecules, generated in response to both endogenous and exogenous stimuli. The most important cellular ROS/RNS include both radical and non-radical oxygen-based molecules, as superoxide anion (O•2-), hydrogen peroxide (H_2_O_2_), hydroxyl radical (•OH), hydroxyl ion (OH−), as well as nitrogen-based molecules including nitric oxide (•NO), nitrogen dioxide radical (•NO_2_) and peroxynitrite (ONOO-) [1,2]. ROS are physiologically produced by NADPH oxidase (NOX), a cell membrane enzymatic complex, and by mitochondria as products of the electron transport chain (ETC), and act as second messengers regulating several signaling pathways [3]. The cellular concentration of ROS is regulated by enzymatic and non-enzymatic antioxidant systems to maintain cellular redox balance. The imbalance between ROS production and antioxidant mechanisms, due to increased ROS production and/or decreased antioxidants activities, results in oxidative stress that promotes protein, lipid and DNA damage [4]. Oxidative stress is considered a common pathophysiological mechanism and it has been associated to many pathological conditions such as cancer [5,6], diabetes [7,8,9,10], cardiovascular [11,12] and neurodegenerative diseases [13,14].

Platelets are anucleated cells derived from megakaryocytes (MKs), which play a crucial role in hemostasis, thrombus formation, atherosclerosis, inflammation and immune response [15,16,17]. Human thrombopoiesis is finely tuned, since the number and function of circulating platelets are essential to maintain hemostasis [18,19,20,21,22,23,24]. Indeed, while excessive platelet release (thrombocytosis) and activation (platelet hyperreactivity) are usually associated with a higher thrombotic risk [25], reduced platelet counts (thrombocytopenia) and altered platelets function, due to inherited or acquired defects [26], might compromise wound healing, resulting in an increased risk of bleeding and hemorragic disorders [27].

**Figure 1:**
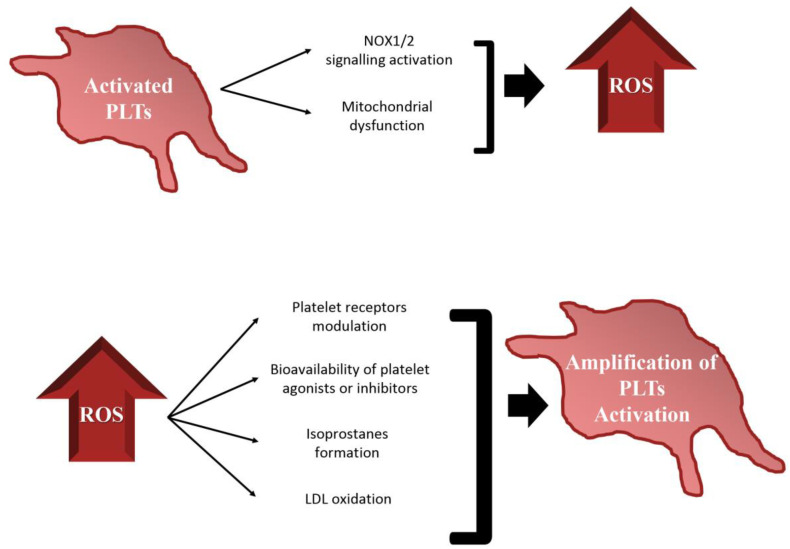
Schematic representation of the relationships between platelet functions and redox state. Activated platelets produce ROS, via NOX1/2 signaling activation and induction of mitochondrial dysfunctions. Intra- or extra-platelet ROS boost platelet activation by regulation of: platelet receptors function, bioavailability of platelet agonist or inhibitors, isoprostanes formation and low-density lipoproteins (LDLs) oxidation.

The pivotal role of ROS and mitochondria in platelet function has recently emerged, regulating platelet activation, aggregation and recruitment, tuning several cellular signaling pathways. [28,29,30]. This aspect is even more relevant if we consider that platelets are both source and target of ROS (Figure 1).

Several studies suggested that MKs differentiation as well as platelet release and activation are modulated by extracellular ROS in a microenvironment-dependent manner [31]. Upon activation, agonists stimulation triggers signaling pathways that result in the production of ROS by both NAPDH oxidases (NOXs) and mitochondria [32,33]. The increased production of endogneous ROS, in turn, alters mitochondria function and boosts platelet activation in an auto-amplifying loop [34].

Despite changes in redox status occur physiologically during platelet activation, the onset of oxidative stress conditions can modify platelet function and lead towards two main pathological outcomes: toxicity and hyperactivation. In turn, toxicity results in excessive platelet apoptosis and therefore in thrombocytopenia and bleeding while, hyperactivation may lead to excessive clot formation and thromboembolic complications.

Oxidative stress has been described in several disorders including atherosclerosis, diabetes mellitus, hypertension, obesity, and cancer [35,36]. In these pathological settings, oxidative stress is the secondary outcome of the diseases; the increased oxidative burden in the circulation exposes platelets to a pro-activatory milieu, responsible for platelet pro-adhesive and pro-aggregatory phenotype, which in turn lead to thromboembolic propensity, a common characteristic of all these diseases. In turn, oxidative stress-activated platelets are a source of ROS, which further contribute to the circulating oxidative stress. This process generates a vicious circle capable to affect other cell types, eventually contributing to diseases progression and complications.

## 2. Sources of ROS in Platelets

Nicotinamide adenine dinucleotide (phosphate) (NAD(P)H) oxidases (NOX) isoforms are the main sources of ROS in platelets, followed by cyclooxygenase (COX), xanthine oxidase (XO) and mitochondrial respiration [37]. NOX enzymes are able to transport electrons from NADPH across biological membranes to reduce oxygen to superoxide (O•2-). The NOX family is composed of seven different isoforms including NOX1, NOX2, NOX3, NOX4, NOX5, DUOX1, and DUOX2; they differ in activation, subunit composition, localization, and expression. All members have six predicted transmembrane domains, motifs for NADPH and FAD (flavin adenine dinucleotide) binding, and conserved paired histidines able to bind to heme groups [38].

In human platelets, both NOX1 and NOX2 significantly contribute to ROS production, and they are considered responsible for the regulation of platelet responsiveness [39]. Both isoforms are inactive in resting platelets and turn on upon stimulation by agonists, forming a multi-complex with several regulatory cytoplasmic subunits. NOX2 forms a multiprotein complex with p22^phox^, p40^phox^, p47^phox^, p67^phox^ and the small GTPase Rac1. NOX1 also interacts with similar proteins, including p22^phox^, NOXO1 (similar to p47^phox^), NOXA1 (similar to p67^phox^), and GTPase Rac1 [40]. The translocation of cytoplasmatic subunits is driven by the protein kinase C-dependent phosphorylation of p47^phox^ and NOXO1; and their subsequent interaction with both p22^phox^ (via a Src homology-3, SH3 domain) and the membrane-associated phosphatidylinositol 3-kinase (PI3K) product, phosphatidylinositol 3,4-biphosphate, PI(3,4)P (via a phox homology PX domain). GTPase Rac1 plays a pivotal role in both NOX1 and NOX2 activation, orchestrating the assembly of the active enzyme complex. Indeed, Rac1-GTP binding to p67^phox^ and to NOXA1 facilitates, in turn, complex formation with NOX2 and NOXA1, respectively, and their functional activation [41]. Recently, small-molecule inhibitors of Rac1–p67^phox^ interaction have been designed in order to prevent ROS production. These small molecules have been validated in vitro and in vivo, and have been proposed as potential anti-thrombotic therapies to reduce platelet hyperactivation and aggregation without affecting the hemostatic response to injury [42].

NOX activity has been correlated to platelet responsiveness in physiological and pathological conditions. Indeed, impaired platelet activation has been described in patients with genetically determined NOX2 deficiency. Pignatelli and colleagues studied platelet activation in X-linked chronic granulomatous disease (X-linked CGD), a rare primary immunodeficiency affecting the innate immunological system, frequently associated with severe infectious diseases. Patients with X-linked CGD, genetically deficient in NOX2 (gp91^phox^), showed defects in platelets, ROS generation and CD40 ligand expression, upon thrombin, collagen, and arachidonic-acid platelet activation [43]. Recently, similar results were obtained in patients with hereditary deficiency of the p47^phox^ subunit, but with milder effects on platelet activation [44]. Treatment of human platelets with non-specific NOX inhibitors phenocopies the effects of ROS scavengers, leading to a significant reduction in intracellular ROS, platelet aggregation and thrombus formation under high shear [45,46].

The differential involvement of NOX isoforms seems to be stimulus-specific, based on the nature of the agonist that triggers platelet activation. Indeed, Delaney and colleagues, comparing NOX1 and NOX2 isoform activation in a knock-out murine model, demonstrated that NOX1 was selectively involved in platelet aggregation and ATP secretion, in response to thrombin and thromboxane A2 analog U46619. By contrast, NOX2 was activated in GPVI/ITAM-mediated platelet activation as well as in GPCR-induced calcium mobilization. NOX2^−/−^ mice had a severe and significant defect in adhesion and recruitment of platelets at the site of laser-induced arteriolar wall injury, suggesting that NOX2, but not NOX1, is involved in thrombus formation [47].

In contrast with this study, Walsh and colleagues suggested that NOX2 had no relevant role in response to collagen, with NOX1 playing the main activating role in the signaling of the collagen-specific GPVI receptor [48]. Recently, the different roles of NOX1 and NOX2 have been investigated using NOX-selective peptide inhibitors and a novel technique to simultaneously monitor platelet activation and oxygen-radical generation [49]. It has been demonstrated that in human platelets, NOX1 is the main source of ROS in response to collagen, while NOX2 is critical for the activation induced by thrombin. Indeed, dismutation of NOX2-derived O•2- to H_2_O_2_ is the main stimulus for platelet-dependent thrombus stabilization. Moreover, both NOX1 and NOX2 are activated by oxidized low-density lipoprotein (ox-LDL) and amyloid-β peptide (Aβ1-42), both capable of modulating platelet function and both associated with the thrombotic complications of atherosclerosis [50] and cerebrovascular amyloid angiopathy (CAA), respectively [51].

NOX-dependent ROS production can, in turn, trigger additional ROS generation by platelet mitochondria. The ubiquinone-binding sites in complex I and III of the ETC have been identified as the main mitochondrial sources of O•2-, which can then be converted into H_2_O_2_ by superoxide dismutase 2 (SOD2) [29]. Mitochondrial ROS production is mainly regulated by the redox state of the ETC, and therefore by mitochondrial membrane potential (∆Ψm) [29]. Hyperpolarization of the mitochondrial membrane is associated with ROS production and platelet activation [52]. Indeed, mitochondria production of ROS observed upon hyperglycemia, a condition often associated with type 2 diabetes mellitus, is due to the increase in ΔΨm and determines platelet aggregation. In this model, the inhibition of ETC complexes completely abolishes ROS production and platelet activation [53]. On the other hand, depolarization of mitochondrial membrane potential is a key step in the procoagulant-platelet microparticle formation [54].

## 3. Platelet Activation Triggers ROS/ Production

In physiological conditions, platelets respond with a functional triade of adhesion, activation and aggregation. Platelet adhesion is mediated by the interaction of platelet glycoprotein VI (GPVI) and GPIbα, with collagen and von Willebrand factor (VWR), respectively [55]. This process triggers platelet activation typified by cytoskeletal and membrane rearrangements, shape change, calcium mobilization activation of the platelet integrin αIIbβ3 (GPIIb–IIIa) [56] degranulation and phosphatidylserine (PS) externalization [56].

The release of adenosine diphosphate (ADP), factor V, fibrinogen, thrombin, and thromboxane A_2_ (TXA_2_) from platelet granules contributes to the amplification of platelet and to the recruitment of additional platelets at the site of growing thrombus. The interaction between fibrinogen and activated integrin αIIbβ3 [57], expressed on activated platelet, induces platelet aggregation and stabilizes the hemostatic plug, through the conversion of fibrinogen to fibrin by thrombin [56].

Oxidative stress and persistent stimuli lead to excessive platelet activation, which typify several pathological conditions including myocardial infarction, stroke, atherothrombosis and metabolic disorders [35].

**Figure 2:**
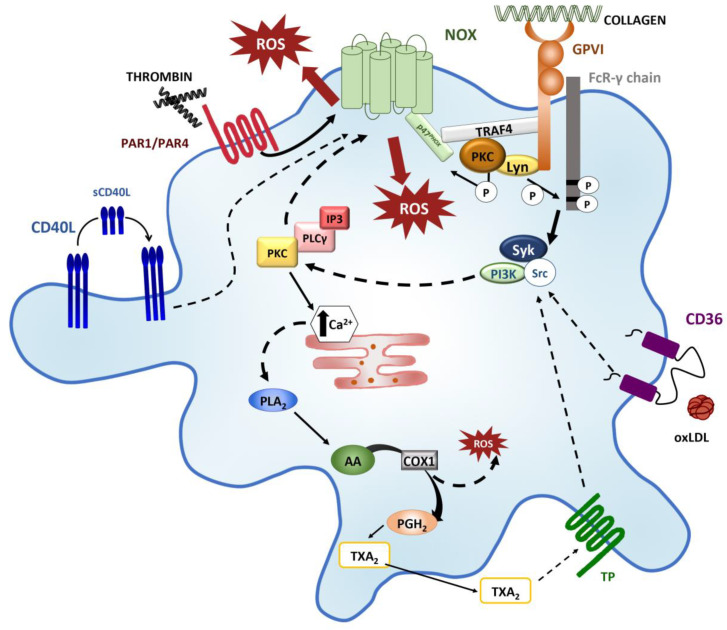
Platelet signaling pathways that trigger ROS production. Collagen binding to GPVI induces ROS production through two distinct Syk-dependent or/and Syk-independent pathways. In the former pathway, TRAF4 and the Src family kinase Lyn, associated with the cytoplasmic tail of GPVI, phosphorylates ITAM sequences activating Syk and PI3K. PI3K phosphorylation leads to (PLCγ2)-IP3/PKCs axis activation, which, in turn, induces NOX-mediated ROS production and Ca2+ mobilization. The increase in intracellular Ca2+ mediates PLA2 activation and the consequent production of ROS by COX1 during AA conversion in TXA_2_. TRAF4 and Lyn are involved also in the Syk-independent pathway, interacting with PKC and activating NOX. Thrombin-PAR1/PAR4 binding promotes NOX activation and ROS release in the extracellular microenvironment. In addition, sCD40L/CD40, ox-LDL/CD36 and TXA_2_/TP interactions trigger signaling pathway that result in NOX-mediated ROS production. (GPVI: glycoprotein VI; Syk: spleen tyrosine kinase; TRAF4 tumor nescrosis factor associated factor 4; ITAM: immunoreceptor tyrosine-based activation motif; PI3K: phosphatidylinositol 3-kinase; PLC: phospholipase Cγ2; IP3: inositol 1,4,5-trisphosphate; PKC: protein kinase C; NOX: NAPDH oxidase; PLA2: phospholipase A2; COX1: cyclooxygenase 1; AA: arachidonic acid; TXA2: thromboxane A2; PAR1/PAR4: protease activated receptors; sCD40L: soluble CD40 ligand; ox-LDL: oxidized low-density lipoprotein; TP: thromboxane receptor).

Platelet intracellular ROS are produced at the basal state by NOX1/2 isoforms, and rapidly increase upon platelet-receptor stimulation (Figure 2).

Bakdash and Williams proposed that ROS production during platelet activation is spatially distinct and dependent on platelet agonist [58]. In the literature, data on this topic are still conflicting. For example, collagen has been found to produce either intracellular [59] or extracellular ROS [46] probably because of its ability to bind and activate multiple receptors. Furthermore, in vitro experiments using convulxin, a GPVI selective agonist, have demonstrated that GPVI-mediated signaling pathway is the main contributor to the intracellular ROS generation [58,59].

GPVI is a type I transmembrane receptor of the immunoglobulin (Ig)-like superfamily, and its cytoplasmatic tail [60] can bind calmodulin (CaM) and Src family kinase, including Fyn and Lyn, key components for the propagation of signals downstream of GPVI receptor [61]. GPVI forms a complex with the Fc receptor γ-chain (FcRγ), that contains an immunoreceptor tyrosine-based activation motif (ITAM). Collagen binding to GPVI/FcRγ complex results in the phosphorylation of ITAM by Lyn and in the activation of spleen tyrosine kinase (Syk) and phosphatidylinositol 3-kinase (PI3K), leading to the activation of phospholipase Cγ2 (PLCγ2)-IP3/PKCs (protein kinase C) signaling pathway. As a consequence, inside-out activation of integrin αIIbβ3 (GPIIb/IIIa) and α2β1 (GPIa/IIa) takes place, mediating platelet aggregation and activation of metalloproteinase-mediated shedding of GPVI [28,62]. Of note, GPVI cytoplasmatic domain might also bind tumor necrosis factor associated factor 4 (TRAF4), which is thought to interact with p47^phox^, a subunit of the NOX1 and NOX2 complex [63].

Two distinct phases of ROS generation have been proposed upon collagen-mediated GPVI activation [63]. The initial phase is Syk-independent: TRAF4 allows the interaction of Lyn with (PKCδ), which, in turn, phosphorylates p47^phox^, inducing NOX-mediated ROS production. TRAF4 can also bind other signaling proteins, such as Hic-5 (focal adhesion adapter protein) and Pyk2 (proline-rich tyrosine kinase 2), which are constitutively associated with Lyn. This TRAF/p47^phox^/Hic-5/Pyk2/NOX axis is thought to be responsible for the rapid burst of ROS [64]. The second phase is Syk-dependent: ligand-induced activation of ITAM leads to Syk activation, which results in the stimulation of the (PLCγ2)-IP3/PKCs axis; lastly, PKCs can activate NOX, supporting the subsequent production of ROS [65].

A similar biphasic mechanism has also been observed in mitochondrial ROS production, in which an initial phase, termed ROS-induced ROS release (RIRR), is followed by a second phase of amplification of ROS release [66].

As mentioned above, PI3K is involved in collagen-induced ROS generation and platelet aggregation. The activation of PI3-K is considered essential for maintaining GPIIb-IIIa and supporting aggregation. Platelets express various PI3K isoforms. The class I PI3Ks, especially PI3Kβ and PI3Kγ, have been widely investigated in arterial thrombosis and cardiovascular disorders, and they are involved in platelet signal transduction during activation [67]. Recently, class III PI3K α (PI3KC2α) has also been identified in this process, playing a critical role in NOX complex assembly and O•2- production, with PI3KC2α^−/−^ mice showing decreased levels of ROS, reduced shear-dependent platelet adhesion and thrombus stability [68].

In contrast to collagen, thrombin seems to be involved in the production of extracellular ROS. Thrombin is a potent platelet mediators that acts via G protein–coupled receptors, as GPIbα and protease activated receptors (PAR1 and PAR4) [69]. Thrombin-stimulated platelets generate extracellular ROS, through the activation of a signaling pathway mainly involving PAR4 and GPIbα binding, and induce NOX1-dependent production and release of O•2-, which further amplifies the initial signals and sustains platelet recruitment and activation [58].

Furthermore, TXA_2_, has also been implicated in platelet ROS production and release [37,45,70]. During the conversion of arachidonic acid into TXA_2_ in activated platelets, cyclooxygenase 1 (COX1) catalyzes the formation of intermediate prostaglandin H_2_ (PGH_2_), and generates ROS as a by-product [71]. Then TXA_2_ is released to promote thrombus growth by recruiting additional platelets. Upon their interaction with TP receptors, TXA_2_ and its synthetic analog, U46619, they increase intracellular Ca^2+^ concentration, which is required for Src phosphorylation and NOX-derived ROS generation. In this model, ROS production is completely abolished with NOX inhibitor apocynin and calcium chelator BAPTA, while it is only reduced in platelets treated with the Abl/Src inhibitor dasatinib, and subsequently stimulated with TXA_2_ agonists [72].

In addition to collagen/GPVI and thrombin/PAR4, NOX2 can be also activated by sCD40L/CD40 and ox-LDL/CD36 interaction. CD40 ligand (CD40L or CD154) is a transmembrane protein stored in platelet α-granules. After platelet activation, CD40L is expressed on platelet membrane and it can be cleaved by metalloproteinases and released into a soluble form (sCD40L) [73] that may activate platelets binding CD40 and αIIbβ3, [74]. sCD40L binding with both receptors induces Akt and p38 MAP kinase phosphorylation. Chakrabarti and coworkers demonstrated that inhibition of NOX decreased the generation of ROS in platelets stimulated with recombinant sCD40L, and identified the Akt-p38 MAP kinase axis as the signaling pathway associated with sCD40L-dependent NOX activation [2].

Ox-LDLs are known to promote platelet hyperactivity and platelet pro-thrombotic phenotype in dyslipidemic disorders and cardiovascular diseases [75]. Ox-LDLs exert their function binding two different platelet receptors: CD36, which is constitutively expressed on platelet membrane, and LOX1 expressed only upon activation. The deletion of these receptors in animal models of artery thrombosis has been associated to a marked reduction in thrombus formation [76,77]. Magwenzi and colleagues have recently demonstrated that ox-LDL/CD36 binding induced the activation of a tyrosine kinase and PKC-signaling that led to NOX2-mediated ROS generation. Ox-LDL-induced ROS generation was markedly reduced by pharmacological inhibition of NOX2 (gp91ds-tat) and completely abolished in CD36^−/−^ and NOX2^−/−^ mice [78].

## 4. Endogenous and Exogenous Antioxidant Systems as Regulators of Platelet Function

Antioxidants play an important role in maintaining redox balance in platelets. Platelets have a number of antioxidant defenses that include antioxidant proteins, which show enzymatic activity, and non-enzymatic molecules able to rapidly neutralize ROS [35]. Enzymatic antioxidants include superoxide dismutase (SOD), catalase (CAT), glutathione peroxidase (GPx), glutathione-S-transferase (GST) and nitric oxide synthase (NOS), while and non-enzymatic antioxidants consist of glutathione, vitamins, carotenoids, polyphenols, and some other molecules [79].

Since platelets, upon activation, produce ROS which, in turn, enhances platelet activation, it is clear that antioxidants can not only prevent the cytotoxic effects of ROS, but can also regulate redox-sensitive signaling pathways in platelets [35]. Antioxidants exert their anti-thrombotic effects by directly converting ROS into more stable molecules, and indirectly increasing NO.

### 4.1. Endogenous Platelet Antioxidant

The first major defense component of the antioxidant system is represented by SOD. SODs are a family of enzymes that catalyzes the dismutation of O•2- in oxygen and H_2_O_2_. SOD family consists of three isoforms with a different cellular localization and metal cofactor: homodimeric Cu/Zn-SOD (SOD1), localized in the cytosol and in the mitochondrial intermembrane space; homotetrameric Mn-SOD (SOD2), localized in the mitochondrial matrix and homotetrameric Cu/Zn-SOD (SOD3) with an extracellular distribution. Platelets express both SOD1 and SOD2. SOD plays an important role in physiological platelet function and in prevention of thrombus formation, promoting endogenous nitric oxide (NO)-bioactivity [80]. In vitro experiments showed that administration of SOD, as well as N-acetylcysteine (NAC), a synthetic inhibitor of ROS, completely abolished O•2- production and collagen-mediated platelet activation. Furthermore, SOD phenocopied the effects of the NOX inhibitor, DPI (diphenyleneiodonium chloride), impairing platelets aggregation [46]. The effects on platelets activation were mainly attributed to the cytosolic SOD1 activity, since SOD2 specially acts on mitochondrial-derived ROS. Recently, Fidler and coworkers demonstrated that SOD2-KO platelets showed increased mitochondrial ROS; however, total platelet ROS content remained unchanged. Furthermore, deletion of SOD2 did not alter tail-bleeding or arterial thrombosis in vivo, suggesting that SOD2 is dispensable for platelet redox balance [81,82].

SOD produces H_2_O_2_ which is a more stable molecule than O•2-; additionally, while O•2- is charged, hardly permeable and short-lived, H_2_O_2_ is uncharged, diffusible and has a longer half-life [83]. H_2_O_2_ may act as a second messenger inducing intracellular calcium mobilization, arachidonic acid and TXA_2_ release and phospholipase C up-regulation in platelets [59].

H_2_O_2_ is efficiently neutralized by both CAT and GPx. Platelets derived from GPx1/CAT double-deficient mice showed elevated cellular ROS levels and enhanced PLCγ2 activation in response to collagen, which subsequently led to increased intracellular calcium levels, degranulation, and integrin αIIbβ3 activation [84].

GPx are a family of selenocysteine-containing enzymes, which use glutathione as a mandatory co-substrate. GPx catalyzes the reduction of hydrogen and lipid peroxide in water and lipid alcohols, oxidizing glutathione (GSH) to glutathione disulfide (GSSG) in the process [85]. Glutathione peroxidase is tightly coupled to glutathione reductase, a NADPH-dependent enzyme, that restores reduced GSH. In platelets, GSH depletion attenuates GPx activity and induces an increase in lipid peroxidation, altering the redox homeostasis [35]. In murine models, GPx deficiency is associated to an increased risk of platelet-dependent thrombosis and vascular dysfunction, due to an impaired ROS metabolism and a decreased NO-mediated platelet inhibition [86].

H_2_O_2_ decomposition could be also catalyzed by the thiol-selenoperoxidase peroxiredoxins. These enzymes and, in particular, peroxiredoxin II (PrxII) negatively regulate various receptor signaling pathways in response to platelet-derived growth factor, epidermal growth factor, or T cell ligands. Recently, the antioxidant function of PrxII has been correlated with platelet activation by Jang’s research group [87]. Indeed, it has been demonstrated that PrxII acts as a negative modulator of GPVI-mediated signaling, by eliminating platelet H_2_O_2;_ depletion of this antioxidant enzyme is associated with a pro-thrombotic platelet phenotype. These results have also been validated in in vivo experiments showing that PrxII-KO mice are more inclined to develop platelet-dependent thrombus formation after carotid artery injury [87].

Reduced/oxidized nicotinamide adenine dinucleotide (NADH/NAD+) and reduced/oxidized nicotinamide adenine dinucleotide phosphate (NADPH/NADP+) couples are closely linked to glutathione (GSH), protein disulphides isomerases (PDIs), thioredoxin (Trx) and PrxII. Indeed, these pyridine nucleotides, take part in ROS generation by NOX, and regulate oxidation/reduction balance. Trx- and glutathione- reductase, for instance, use NADPH as an electron donor to convert the oxidized forms of Trx and glutathione (GSSG) into their reduced states. The NADPH/thioredoxin reductase/thioredoxin system regulates the thiol profile of key platelet adhesion/activation receptors and influences platelet reactivity to collagen (GPVI-pathway) and von Willebrand factor (GPIb-IX-V-pathway) [88].

Glutathione is the main non-enzymatic antioxidant within cells. Glutathione presents a redox-active thiol group thiol group [sulfhydryl (-SH) group in the cysteine residue], that becomes oxidized (GSSG) when GSH reduces target molecules. The extra- and intracellular ratio of the reduced form (GSH) to the oxidized form (GSSG) is indicative of oxidative stress in various cells [89]. GSH exerts its antiplatelets effects regulating platelet TXA_2_ synthesis [90], and enhancing the antiplatelet activity of S-nitrosoproteins. Low GSH/GSSG ratio has been associated with greater susceptibility of platelet to activating agents [91].

### 4.2. Exogenous Redox Systems

Platelets may be also affected by the surrounding microenvironment, including blood cells, vascular endothelial cells, vascular smooth muscle cells and fibroblasts. In physiological conditions, resting state of platelets is preserved by endothelial production of •NO and prostacyclin [92]. Endothelial- and platelet-derived •NO is also essential for limiting platelet activation and thrombus growth following tissue damage, and recovering normal homeostasis. In pathological conditions or under oxidative stress, vascular endothelium, activated macrophages, neutrophils and damaged erythrocytes release elevated amounts of ROS, which boost proinflammatory and procoagulant platelet functions. •NO antiplatelet effects are impaired upon the reaction with O•2-, which generate peroxynitrite.

Vascular antioxidants systems (SOD, CAT, GPx, glutathione S-transferases, Prx and heme oxygenase 1, HO-1) and extracellular antioxidant compound, as GSH/GSSG and GPx3, could directly or indirectly, contribute to limit the ROS/RNS-mediated oxidative reactions in platelets and to restore the redox balance [93]. Extraplatelet SOD and CAT inhibit αIIbβ3 activation and P-selectin expression after thrombin stimulus. The extracellular isoform of GPx, GPx-3, can scavenge reactive oxygen species in the extracellular compartment and enhance •NO bioavailability [94]. Moreover, in the murine model, GPx-3 deficiency has been associated with platelet-mediated arterial thrombosis [95]. Recently, the plasmatic GSH/GSSG ratio has emerged as a regulator of platelet function, controlling the activation of platelet membrane PDIs (platelet disulfide isomerase). Indeed, the balance between dithiol and disulfide fractions at the active site of PDI, regulates the activation of platelet GP IIb/IIIa and GPIb integrins, ensuring high-affinity binding [93].

In addition, exogenous antioxidants derived from the diet including vitamin E, vitamin C, carotenoids, some minerals (Zn, Mn, Cu, Se) and polyphenols (flavonoids, phenolic acids, stilbenes, lignans), act as non-enzymatic scavengers preserving redox balance and regulating platelet functions [79]. These antioxidants are present in fruits, vegetables, commonly consumed beverages (juices, tea, coffee, wine), extra virgin olive oil, nuts, cereal products and cocoa [96]. Many epidemiological studies have pointed out that a diet rich in polyphenols reduces the susceptibility to various diseases, such as diabetes, and Alzheimer’s disease [97,98], and they have been associated with a low risk of thrombotic events and cardiovascular mortality [99]. The beneficial effects of polyphenols are in part related to their antioxidant and anti-thrombotic properties. Polyphenols in cocoa and extra virgin olive oil can regulate platelet function, as demonstrated by the inhibitory effect of polyphenol-rich nutrients on platelet activation [100]. Catechin and epicatechin from cocoa and polyphenols from extra virgin olive oil exert their antiplatelet effect through the down regulation of NOX2 and the consequent reduction in the formation of ROS [101,102,103]. Polyphenols could also enhance •NO generation and/or bioactivity, which leads to inhibition of platelet activation and could trigger the Nrf2-Keap1 (nuclear factor erythroid-2 related factor 2/kelch-like ECH-associated) axis, which in turn leads to the activation of the antioxidant response element [104]. In addition to polyphenols, vitamin E (alpha-tocopherol) and vitamin C (ascorbic acid) have anti-platelet and anticoagulant properties [105]. In particular, the antiplatelet activity of vitamin C is mainly due to its capacity of quenching superoxide radicals [106].

## 5. Redox Control of Platelet Activation

**Figure 3:**
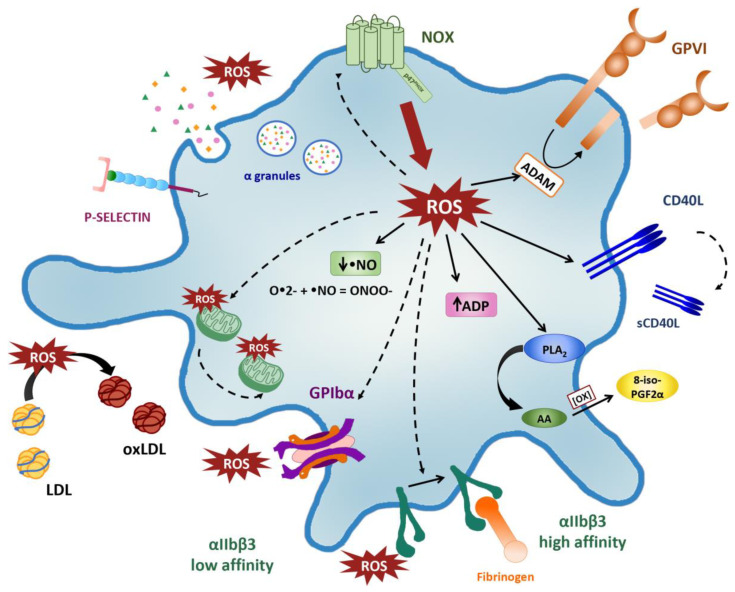
Redox control of platelet activation. NOX-derived ROS boost further ROS production and the consequent platelet activation, adhesion and recruitment in an auto-amplifying loop. Intraplatelet and extracellular ROS regulate platelet receptor (GPIbα and αIIbβ3) binding affinity and GPVI and GPIbα shedding via ADAMs. NOX-derived ROS also regulate ADP and •NO bioavailability, α granules release and P-selectin exposure on platelet membrane. ROS potentiate the PLCγ/PKC/MAPKp38 signaling cascade, thereby inducing PLA2 activation and 8-iso-PGF2α formation. ROS upregulate CD40L surface expression and release in response to platelet agonists. Upon secretion in the vascular lumen, platelets-derived ROS promote the oxidation of circulating LDL. NOX-derived ROS induce mitochondrial dysfunction. (NOX: NAPDH oxidase; GPIbα: glycoprotein Ibα; αIIbβ3: glycoprotein GPIIb/IIIa; GPVI: glycoprotein VI; ADAM: disintegrin and metalloproteinase; ADP: adenosine diphosphate; NO: nitric oxide; PLCγ: phospholipase Cγ2; PKC: protein kinase C; MAPKp38: P38 mitogen-activated protein kinases; PLA2: phospholipase A2; 8-iso-PGF2α: 8-iso-prostaglandin F2α; CD40L: CD40 ligand; LDL: low-density lipoprotein).

Platelet-derived ROS act as second messengers and can influence different signaling pathways which enhance agonist-induced platelet function. Although the exact molecular mechanisms by which ROS affect platelet function are still under investigation, NOX-derived ROS have been suggested to regulate: (i) platelet receptor activity; (ii) bioavailability of both platelet agonists and inhibitors; (iii) isoprostane formation; (iv) LDL oxidation (Figure 3).

Oxidative changes occurring inside platelets after activation seem to modulate platelet receptor function, including αIIbβ3, GPIbα and GPVI [45,107,108,109].

The αIIbβ conformational change, leading to fibrinogen binding and platelet-platelet interaction, is blocked by NOX inhibitors and superoxide scavengers, though a NO/cGMP-independent pathway [45]. The role of platelet-derived extracellular ROS in integrin αIIbβ3 activation is still unclear. It has been proposed that extracellular ROS can interact with thiol groups present in the extracellular domain of αIIbβ3, promoting integrin activation [93]. These data suggest the direct role of redox systems in thrombin-induced αIIbβ3 activation. Moreover, in a recent study by Kim et al., platelet NOX2-produced ROS regulated P-selectin exposure upon thrombin stimulation and ligand-binding function of GPIbα. ROS induced the oxidation of GPIbα sulfhydryl groups promoting platelet adhesion and platelet–leucocyte interaction [107].

Furthermore, ROS are involved in the mechanism of platelet receptor shedding that reduces platelet adhesive capacities and results in platelet dysfunction. However, it has been recently demonstrated that platelet receptor shedding increase coagulation factor binding and enhance thrombin and fibrin generation, which result in a platelet procoagulant phenotype [110]. Platelet activation is closely associated to GPVI and GPIbα shedding and ectodomain release in plasma. This event is mediated by the activation of metalloproteinases (ADAMs). ADAMs are a family of disintegrins and metalloproteinases that catalyze the cleavage of the ectodomains of GPVI and GPIbα. In particular, it is considered that ADAM17 primarily cleaves GPIbα, whereas ADAM10 predominantly acts on GPVI [111,112]. Interestingly, ROS can oxidize cysteine residues located on the cysteine-rich domains of ADAMs and/or interact with intracellular cytoplasmic domains of these metalloproteinases, promoting their activation [113]. Furthermore, ROS induce GPIbα shedding trough the oxidation of cysteine residues of different protein kinases including p38-MAPK, known to active ADAM17 [109]. Recently, Hosseini and coworkers demonstrated that receptor shedding is partially inhibited when activated platelets are treated with reducing agents such as NAC or DTT, suggesting a role of ROS in this process [113].

ROS are also thought to regulate GPVI signaling cascade in a redox-dependent mechanism, and to consequently boost NOX related ROS production. Indeed, ROS produced upon collagen stimulation, prevent Src homology region 2-containing protein tyrosine phosphatases 2 (SHP-2) inhibition of GPVI signaling through the direct oxidization of the catalytic cysteine of SHP-2 [84].

Platelet-derived O•2- upregulates CD40L surface expression and release in response to platelet agonists, as thrombin and convulxin. On the contrary, antioxidants (such as extracellular SOD and vitamin C) as well as NOX inhibitors significantly inhibit CD40L upregulation [114]. Similarly, in patients with an inherited deficiency of gp91^phox^ (the catalytic core of NOX) the expression of O•2- and CD40L expression by activated platelets have been found almost completely suppressed [43]. However, the molecular mechanism(s), that underlie intra- and extra-cellular ROS effects on CD40L, remains unknown.

In addition to CD40L upregulation, ROS have been found to modulate platelet α-granule exocytosis. Indeed, Bakdash and colleagues demonstrated that NAC and synthetic antioxidant compounds significantly inhibited surface membrane expression of CD62p and RANTES release in thrombin activated platelets [58].

ROS regulate the bioavailability of platelet agonists and inhibitors as ADP and •NO. Platelet-derived O•2- has been demonstrated to support platelet recruitment by increasing ADP. Collagen stimulation induces a strong release of ADP, but the amount of ADP in supernatants of collagen-stimulated platelets were decreased in presence of SOD. Therefore, Krotz and coworkers suggested that collagen-induced O•2- may inactivate platelet ADP-destroying ectonucleotidase and thereby extend ADP availability and function [46]. •NO produced by both endothelial cells and platelets is a potent inhibitor of platelet aggregation through the activation of a NO-sensitive guanylyl cyclase (NO-GC) [115]. The O•2- can rapidly react with •NO to form ONOO-, decreasing •NO availability and thereby abolishing its antiplatelet function. The role of NOX in counterbalancing •NO activity was recently confirmed in patients with chronic granulomatous disease in which NOX genetic deficiency determined complete suppression of platelet O•2- production and increased levels of •NO [116].

Intra-platelet ROS contribute to lipid peroxidation that leads to the formation of isoprostanes. The 8-iso-PGF2α is considered a gold-standard biomarker of oxidative stress, and elevated levels have been found in the plasma and urine of patients with cardiovascular disorders [117,118,119], atherosclerosis [120,121], type 2 diabetes mellitus [122,123] and hypertension [124]. Upon stimulation, platelets release 8-iso-PGF2α primarily via nonenzymatic oxidation of AA catalyzed by NOX-derived O•2-. Accordingly, NOX2-deficient patients with CGD show an impaired ROS production and a decrease in platelet 8-iso-PGF2α levels. A similar inhibition of isoprostanes was described in platelets treated with the NOX2 inhibitor apocynin [116].

Finally, platelet-derived ROS, secreted in the vascular lumen, are involved in the oxidation of circulating LDL. High plasma concentrations of ox-LDL have been found in patients with Type 2 diabetes mellitus as consequence of hypercholesterolemia and hyperglycemia, and they have been correlated with cardiovascular events [125,126]. Recently, activated platelet have been demonstrated to promote in vitro LDL oxidation; indeed, incubation of activated platelets with both purified LDL and homogenized atherosclerotic plaque resulted in a marked increase in ox-LDL levels. The oxidation of LDL was significantly inhibited by NOX specific inhibitors and aspirin and was not observed in platelet of CGD patients, suggesting the involvement of NOX2 and ROS in this process [127].

## 6. Role of Platelet Mitochondria in Redox Balance

Platelets are considered the most metabolically active circulating cells under basal conditions [128] and mitochondria have been considered for decades as the main energy source to support platelet function on platelet function.

In resting platelets, approximately 60% of ATP is provided by glycolysis while only the remaining 40% is produced during the oxidative phosphorylation (OXPHOS) [128]. Platelets ability to promptly respond to stressors or agonists, is associated to an extraordinary energy demand. Therefore, platelet activation seems to markedly alter platelet metabolism, resulting in integrated energetic response to both glycolysis and OXPHOS [129].

Platelet activation experiments, performed in hypoxic conditions, have demonstrated that oxidative energy is essential to guarantee optimal platelet functionality, and anaerobic glycoysis only partially compensates impaired OXPHOS [130,131,132]. Furthermore, Tomasiak et al. showed that alterations of mitochondrial complex III (cytochrome oxidase) or mitochondrial complex IV, mediated by NO, reduce mitochondrial ATP production, resulting in the inhibition of platelet aggregation and secretion [133].

However, mitochondria are not only involved in energy supply, but they play a crucial role in platelet activation and apoptosis, regulating redox balance. Indeed, OXPHOS inevitably leads to mitochondrial O•2- production and release, mainly by complex I and III of the ETC, thus rendering mitochondria themselves an important source of ROS [134]. Mitochondrial O•2- can be then converted in H_2_O_2_ by SOD2 [29]. Different research groups have demonstrated that platelet activation by collagen and thrombin induces a rapid and transient increase in the mitochondrial membrane potential (ΔΨm) and OXPHOS, likely via Ca^2+^ mobilization [135,136]. The increase in ΔΨm is associated to the increased production of ROS in mitochondria, and hyperpolarization of the membrane reduces the electron transport chain, resulting in a leakage of electrons from the chain followed by promotion of the production of O•2- [137]. Furthermore, hyperglycemia plus collagen has been related to the hyperpolarization of platelet mitochondria, resulting in ROS generation and subsequent activation. In platelets treated with inhibitors of mitochondrial complex II and uncouplers of OXPHOS, the increase in mitochondrial ROS, induced by hyperglycemia plus collagen, is completely prevented [53].

Interestingly, a biphasic process, named ROS-induced ROS release (RIRR), has been described in mitochondrial ROS production, characterized by an initial phase of slow release and a second phase of amplification of ROS release [66].

This process has been studied in cardiomyocytes, smooth muscle cells and endothelial cells [138,139].

Indeed, analyzing mitochondrial ROS production kinetics, initial ROS (the so-called “trigger ROS”) are produced by OXPHOS—rapidly engaged to match metabolic needs—and released in the cytoplasm. Through a positive-feedback mechanism, ROS trafficking between mitochondria results in an elevated production of ROS, responsible for the oxidation of essential mitochondrial components. The consequent ROS burst and release is associated with the collapse of the ΔΨm and the formation of mitochondrial permeability transition pores (mPTP) in a calcium-independent manner [140]. Mitochondria exhibit a sort of “ROS excitability” and can respond to either exogenous or endogenous ROS, by increasing their own ROS production in a self-promoting cycle [66]. A similar crosstalk was also observed between mitochondrial ROS and NOX-derived ROS in several cellular models. In human 293T cells transient ROS production by mitochondria stimulated PI3K which promote translocation of Rac1 to NOX1 complex and ROS generation [141]. In endothelial cells [142] and in cardiomyocytes [143], angiotensin II stimulation activated NOX by a PKC-mediated mechanism; NOX-derived ROS, in turn, decreased ΔΨm leading to mitochondrial ROS formation. It is possible to speculate that similar processes occur also in platelets during activation, but the molecular mechanisms and signaling pathways involved in RIRR and in the crosstalk between NOX and mitochondria need to be further investigated.

Platelet activation, ROS increase and the subsequent mitochondrial dysfunction lead to platelet apoptosis, which has therefore been considered a clearance strategy to eliminate hyper-activated platelets from the bloodstream/circulation, and it was recently associated with thrombocytopenia in pathological conditions [144]. It has been demonstrated that low concentrations of platelet agonists induce only an ‘apoptotic-like events’ [145]. On the contrary, potent platelet agonists, such as thrombin and ionomycin, determine the formation of mPTPs which results in a drastic depolarization of mitochondrial membrane and increase in H_2_O_2._ Indeed, mPTPs are nonselective multiprotein pores that cross the inner and outer mitochondrial membranes and cause a rapid collapse of ΔΨm, due to the impaired proton shift towards the mitochondrial intermembrane space. The formation of mPTPs plays a key role in the regulation of platelet activation, inducing platelet transition from an “activated” to a “highly activated” state typified by vesiculation, high-level phosphatidylserine (PS) externalization and high-level fibrinogen retention [146]. Furthermore, mPTPs and ROS are associated to the activation and the translocation of pro-apoptotic protein Bid, Bak and Bax to the mitochondria. These effects evoked by thrombin are significantly attenuated by catalase, indicating the central role of ROS in platelet apoptosis [136,147].

**Figure 4:**
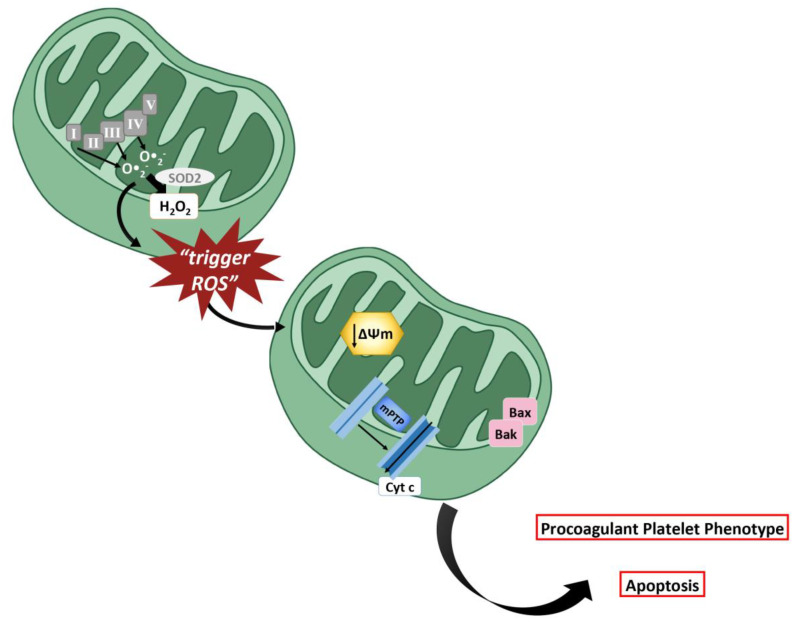
Platelet mitochondria in redox balance. During oxidative phosphorylation, mitochondria produce ROS. Complex I and III of ETC (electron transport chain) are the main sources of mitochondrial ROS, called trigger ROS. Trigger ROS are released in the cytoplasm and, through a positive feedback resulted in an elevated production of ROS. The ROS burst and release induce the collapse of the mitochondrial membrane potential (ΔΨm) and the opening of mitochondrial permeability transition pores (mPTP). These latter events lead to high-level phosphatidylserine externalization, high-level fibrinogen retention, release of cytochrome c, activation and translocation of pro-apoptotic protein Bak and Bax, contributing to platelet procoagulant phenotype and platelet apoptosis.

Of note, ROS have been suggested to directly alter mitochondrial membrane permeabilization leading to the release of proapoptotic factors, such as cytochrome C and caspases, into the cytosol. Indeed, in hyperthermia-induced platelet apoptosis, increased mitochondrial ROS easily oxidize cardiolipin, an important component of the inner mitochondrial membrane, promoting mitochondrial translocation of Bax, cytochrome-C release, caspase-3 activation, PS exposure and ΔΨm depolarization [148] (Figure 4).

## 7. Methods to Assess Platelets Redox Biology

The crucial role of platelets and oxidative stress in cardiovascular disorders and inflammatory diseases has prompted the research of innovative methods to analyze platelet redox systems, as well as in order to monitor disease and therapy [128,149].

### 7.1. Detection of Reactive Oxygen Species Levels

Despite the increasing interest in this field of platelet biology, the development of highly accurate and specific techniques is still challenging because of the evanescent nature of ROS. ROS half-life varies from approximately 10–9 s for highly reactive •OH, to 10–5 s for H_2_O_2_ [150]. Different methods have been established that allow the direct or indirect measurement of redox states in platelets, including spectrophotometry, chemiluminescence, electron spin resonance and spin-trapping [151]. These techniques are too complex and laborious for routine testing. Therefore, an accurate and standardized method for platelet ROS detection represents an unmet need with rapid potential clinical transferability. Here, we briefly present the up-to-date methods for platelet ROS and redox balance assessment, discussing their advantages and limits in both experimental and clinical settings.

Flow cytometry (FCM) is the most widely used technique to measure intracellular levels of ROS. Due to its high versatility and reliability, FCM represents the candidate technique for platelet studies. Thus, FCM can be feasibly employed in research and clinical practice to monitor the response to anti-aggregation therapies, and to assess thrombotic risk in cardiovascular diseases [26,152,153,154,155,156].

The main advantages of this technique are: (i) the requirement of limited sample volumes (only 5 μL); (ii) the possibility to perform the analysis either on freshly isolated platelets, whole blood or platelet concentrates, with minimal sample manipulation [26]; (iii) the ability of simultaneous single-shot analysis of several parameters including platelet phenotype, function, activation markers, platelet–monocyte and platelet–leucocyte interaction, and apoptosis.

Concerning platelet ROS, several fluorescent probes have been developed, with different degrees of specificity and sensitivity [157,158] (Table 1).

Specific kits are commercially available to monitor ROS levels in untreated platelets, in platelets stimulated agonists or/and treated with NAC.

In general, these probes are non-fluorescent compounds which can passively diffuse into cells and, upon interaction with intracellular ROS, are converted into fluorescent compounds. The fluorescence signal is proportional to cellular ROS levels [169].

Dichlorodihy-drofluorescein diacetate (DCFH-DA) is commonly used for detecting H_2_O_2_ in cells and in platelets. Once in the cell, it is hydrolyzed by intracellular esterases to DCFH which remains trapped inside the cells. DCFH is oxidized by ROS/RNS, generally H_2_O_2_, becoming the fluorescent 2′,7′-dichlorofluorescein (DCF) detectable by FCM [163]. Dihydroethidium (DHE) has been suggested as an alternative to DCFDA [165]. The detection of DHE oxidation derivates is generally performed by liquid chromatography combined with mass spectrometry (LC–MS) [78]. Abubaker and colleagues have recently developed and validated an alternative DHE probe-based technique for the detection, by FCM, of O•2- in platelets. This technique is based on the intracellular detection of 2-hydroxy-ethidium (2OH-Et+), the product of DHE oxidation by superoxide anions. The generation of 2OH-Et+, which has an excitation peak at around 400 nm wavelength with emission at 580 nm, can be monitored by FCM but also by confocal imaging and live imaging [166]. Robinson and colleagues have reported that mitochondrial O•2- can be accurately quantified in live cells using FCM [167]. MitoSOX is the mitochondrion-targeted form of DHE. Due to the positive charge of the cationic triphenyl phosphonium substituent, MitoSOX is rapidly targeted to the mitochondria, where it is oxidized by superoxide to form 2-hydroxymitoethidium, which is excited and emits at 510 and 580 nm, respectively [167,168].

Furthermore, intracellular NO can be monitored by FCM with DAF-FM (4-amino-5-methylamino-2′,7′-difluorofluorescein) diacetate, a pH-insensitive fluorescent dye that emits fluorescence after reaction with an active intermediate of •NO, formed during the spontaneous oxidation of •NO to NO2- [161]. Although DAF-FM has been primarily utilized to assess •NO production by endothelial cells, it has been recently used to monitor •NO production by endothelial-like NO synthase (eNOS) in platelets [162].

FCM has also been applied to lipid peroxidation detection. The 4,4-difluoro-5-(4-phenyl-1,3-butadienyl)-4-bora- 3a,4a-diaza-s-indacene-3-undecanoic acid (C11-BOD- IPY581/591) is the main lipophilic dye used for lipid oxidation by oxyl-radical in leukocytes and platelets [160]. Due to its lipophilic properties, C11-BOD- IPY581/591 passes through cell membrane and, once inside, the polyunsaturated butadienyl portion of the dye is oxidized by HO• or ROO• together with the endogenous fatty acids. When excited by blue light at 488 nm wavelength, the molecule has a constitutive fluorescence emission with a maximum at 595 nm, but after oxyl-radical induced oxidation by HO• or ROO•, the fluorescence emission shifts from red to green with a maximum emission at 520 nm [159].

Several methodological limitations should be considered for FCM application in platelet ROS detection. Basal levels of ROS should be measured only in fresh cells, as soon as possible, because of the extremely low stability of ROS. Time-lapse from blood sampling, sample transport and isolation may influence the results. Furthermore, the majority of probes are not designed to react with only a specific type of ROS, and they often react with more than one ROS. For instance, DCFH is oxidized by H_2_O_2_, but it can also interact with HO•, ROO•, •NO and ONOO−. DCFH oxidation can also be promoted by Fe2+ in the presence of O_2_ or H_2_O_2_. It is also important to consider that some FCM probes detecting ROS may also be sensitive to NO (for instance CellROX^®^ Orange), which inhibits platelet aggregation. Results could also be influenced by the experimental setting, such as incubation temperature, probe concentration and uptake by the cell. Additionally, the presence of intracellular probes per se could reduce platelet activation and ROS levels: elevated concentrations of probe may induce cell morphology changes and artifacts in platelets. For example, an elevated concentration of MitoSOX can overload and impair mitochondrial function [167].

Some probes, such as DHE and DFCH-DA, require a two-step reaction to detect ROS. DFCH-DA do not interact directly with H_2_O_2_, and must first be hydrolyzed by intracellular esterases. Therefore, the presence of plasma esterase and esterase inhibitors [164] could potentially interfere with probe staining and compromise the analysis of whole blood and platelet-rich plasma (PRP). In addition, DHE and MitoSoX-based reactions implicate the formation of free-radical intermediates that can potentially be a substrate for reaction with the antioxidants or induce a redox-cycling mechanism that leads to an artificial amplification of the fluorescence signal [151].

Other potential approaches have been proposed to detect extracellular ROS. Some research groups used a chemiluminescence method to detect platelet-released O•2-. Lucigenin (N,N′-Dimethyl-9,9′-biacridinium dinitrate) and the luminol analog L-012 are the commonly used chemiluminescent dyes. Superoxide levels might be artificially overestimated with these dyes, due to the redox phenomenon in which lucigenin and L-012 radicals react with O_2_ to further generate O•2- [37,170].

Platelet O•2- release can be evaluated by cytochrom-C assay. This method is based on reduction of ferricytochrome C by O•2- to ferricytochrome C which is measured monitoring the absorbance at 550 nm using a spectrophotometer [46,151]. The assay must be performed in the presence and in the absence of SOD to determine the SOD-inhibitable signal and avoid artifacts. Since ferricytochrome c is a large protein unable to pass through the cell membrane, this method is not suitable to detect O•2- in the cytoplasm or in the mitochondria of intact cells [46,151].

Recently, Vara and coworkers developed a novel technique to simultaneously monitor platelet activation and oxygen-radical generation. This multi-parametric analysis combined turbidimetry (for platelet activation) with electron paramagnetic resonance (EPR) or EPR spectroscopy (for oxidative status), and it could potentially find applications in clinical practice [49].

### 7.2. Detection of Antioxidant Enzymes Activity

Another common method, used to determine cellular and platelet redox state, is based on the evaluation of ROS-generating and antioxidant enzyme profile, considering transcript and protein levels by both RT-PCR and Western blot analysis [171]. As mentioned above, NOX and XO are the main ROS sources in platelets, while the most important antioxidant enzymes are SOD, CAT, and glutathione-dependent enzymes, such as GPX, GR, and glutathione transferases (GSTs) [35]. Since mRNA and protein do not necessarily reflect enzymes activity, antioxidant enzyme expression must be accompanied by enzyme biochemical activity evaluation.

Several assays, with high sensitivity, are now available and they are mainly based on colorimetric reactions whose products can be estimated by absorbing the fluorescence relatively quickly. However, methods based on more sophisticated tools, such as high-performance liquid chromatography, have been developed [172]. SOD activity can be determined by a direct method based on H_2_O_2_ production measurement [173] or by a spectrophotometric assay, which involves superoxide generation by a xanthine/XO enzymatic system, superoxide-dependent reduction of cytochrome c or tetrazolium salt WST-1 by O•2- to yellow colored formazan, and dose-dependent inhibition by SOD [174]. SOD activity can also be detected semi-quantitatively using an in-gel activity assay, which employs a redox-sensitive dye NBT as a detector of O•2-, and a non-enzymatic superoxide-generating photochemical reaction, combined with polyacrylamide gel electrophoresis [175].

CAT-mediated H_2_O_2_ reduction can be measured by different colorimetric or spectrophotometric assays, that measure the amount of unconverted H_2_O_2_ reacting with an OxiRed^TM^ probe to generate a final product. The change in H_2_O_2_ concentration is directly monitored following the decay in absorbance at 240 nm [176]. GPX activity can be measured using cumene hydroperoxide and GSH as substrates in a coupled reaction with GR. The GSSG formed during this reaction is converted to the reduced state by GR in the presence of NADPH. The oxidation of NADPH, which is proportional to the activity of GPX and GR, can be monitored spectrophotometrically at 340 nm [177].

Furthermore, several assays have been proposed to assess GSH levels and GSH/GSSG ratio in biological samples (spectrophotometry, HPLC, capillary electrophoresis, nuclear magnetic resonance, and mass spectrometry). In platelet extracts, GSH /GSSG ratio is often evaluated using the GSH reductase enzyme method. This assay consists of the thiol-mediated conversion of 5,5′-dithio-bis (2 nitrobenzoic acid) (DTNB; Ellman’s reagent) to 5-thio-2-nitrobenzoic acid (TNB), monitored spectrophotometrically at 412 nm. TNB formation is proportional to the concentration of GSH in the sample [178].

### 7.3. Detection of Protein Oxidation Products

Proteins represent a wide target for ROS, therefore protein oxidation has been considered an indirect marker of oxidative stress. Several amino acidic residues can undergo oxidative modifications including oxidation of sulphur-containing residues, hydroxylation of aromatic groups, nitration of tyrosine residues, nitrosylation and glutathionylation of cysteine residues, conversion of some amino acid residues to carbonyl derivatives. Several methods have been developed for the detection of the different kinds of protein modifications; however, the ability to identify and quantify specific protein oxidative modifications is still limited [159].

Given the relative stability of *carbonylated proteins*, the measure of carbonyl levels in proteins is the most widely used marker of oxidative protein damage. Enzyme-linked immunosorbent assay (ELISA) and HPLC are the most used methods for these purposes and the detection of protein carbonyl groups generally involves the derivatization of the CO group with 2,4-dinitrophenylhydrazine (DNPH) with the formation of a stable dinitrophenyl (DNP) hydrazone product. DND can be detected by several methods which include the direct spectrophotometric measurement of DNP adducts, as well as more specific techniques based on anti-DNP antibodies, like ELISA, Western blot after one-dimensional or two-dimensional electrophoretic separation, immunohistochemistry, and HPLC [159]. The increase in protein carbonylation has been reported in activated platelets and in platelet concentrates undergoing platelet storage lesion [171]. Moreover, Alexandru and co-workers demonstrated that H_2_O_2_ produces dose-dependent increase in the carbonylation of platelet proteins (vs. basal condition) and that thrombin activation stimulates protein carbonyl formation in a process quenched by antioxidant catalase [179].

*3-nitro-tyrosine* (3-NO-Tyr) is the main product of tyrosine oxidation and can be generated through several pathways that include the reaction with ROS and RNS such as ONOO− and NO2 • [180,181,182]. 3-NO-Tyr detection requires gas or liquid chromatographic techniques coupled to mass spectrometry; moreover, ELISA assays based on specific antibodies are also available, despite their use being limited by the variable affinity of antibodies to different nitrated proteins and the low sensitivity [183]. Very few studies have measured platelet nitrotyrosine expression. Specifically, an increase in platelet nitrotyrosine has been reported in a canine model of acute coronary syndromes [184] and in coronary heart disease patients, but not in healthy donors, after ONOO- treatment [185].

### 7.4. Detection of Lipid Peroxidation

In addition to the described methods, the analysis of lipid peroxidation represents a different and valid approach. Lipid peroxidation is widely used as a marker of oxidative stress in various cells, including platelets. The increased generation of ROS may induce enhanced lipid peroxidation of cell membrane phospholipids or circulating LDL leading to the increased generation of F2-isoprostanes, a family of prostaglandin isomers produced from arachidonic acid by a mechanism catalyzed by free radicals [186,187]. Lipid peroxidation, triggered by ROS, is an autocatalytic chain reaction, which catalyzes the hydrogen subtraction at the unsaturated bonds generating a carbon-centered fatty radical that can further react with oxygen producing a lipid peroxyl radical. Lipid peroxyl radicals induce the formation of lipid hydroperoxides which, in the presence of transition metals, generate lipid alkoxyl and ROO• as well as HO•. These products can further sustain the oxidation chain and determine the production of malondialdehyde (MDA) and 4-hydroxy-2-nonenal (HNE). The lipid peroxyl radicals and final products of this process are both known to inhibit protein synthesis and alter enzymatic activity and chemotactic signals [188]. HNE can be detected by high-performance liquid chromatography (HPLC), gas chromatography coupled with mass spectroscopy (GC-MS), and immunological techniques using specific anti-HNE antibodies. The thiobarbituric acid assay (TBA test) is a widely employed procedure to assess lipid oxidation. This assay is relatively simple, does not require technical skills and it can be applied both on fresh and long-term stored platelets. The TBA test is based on the reaction of MDA with thiobarbituric acid (TBA) which generates a pink adduct complex, easily quantifiable by a colorimetric or fluorometric assay. Hemolysis and TBA unspecific reaction with other several compounds can markedly compromise the assay, producing artifacts and overestimating MDA measurement. Moreover, hemolysis can falsely increase the measured MDA levels. Butyl hydroxytoluene (BHT) is usually added to cell lysate in order to prevent further MDA generation during the procedure [189].

Another reliable marker of oxidative stress is represented by 8-isoprostane. 8-isoprostane is the best characterized compound belonging to the F2-isoprostanes formed by free radical peroxidation of biomembranes and then released in free form by phospholipase action. The reliable detection of 8-isoprostane in whole blood and platelet-rich plasma requires gas/liquid chromatography coupled with mass spectroscopy techniques (HPLC/GC-MS) and it is affected by hemolysis. Immunoassay techniques, based on specific antibodies, are under development, but their application is still limited [159].

### 7.5. Analysis of Mitochondrial Function

Mitochondrial dysfunction is often associated to oxidative-associated disorders and inflammatory diseases [190,191,192,193,194,195]. Since platelets represent an accessible source of mitochondria, several research groups have widely investigated bioenergetic platelet profile which might have potential clinical applicability as a diagnostic and prognostic tool as well as a biomarker in treatment monitoring.

Many biochemical assays are available to determine the contribution of the mitochondrial ETC to platelet activation. These assays include the expression analysis of the respiratory chain complexes by Western blot or RT-PCR, measurement of the mitochondria membrane potential activity, ATP content assay, and cytochrome c oxidase (complex IV) and succinate dehydrogenase (complex II) activity using immunoassay or widespread spectrophotometric methods [196]. However, the evaluation of the isolated complex expression and activity does not offer accurate data on the global respiratory function, because ETC complexes are closely linked and depend on each other. In addition, these assays cannot be performed on intact cells and in real time.

Intracellular oxygen concentration and oxygen consumption together with the other markers, such as ATP content, mitochondrial membrane potential, reflect the respiratory activity and the bioenergetic status of cells.

These parameters can be determined in platelets using a Clark oxygen electrode. This method has been used with isolated mitochondria and platelets but showed several problems including uniform signal drift, low sensitivity, changes in sensor response, and bubble formation on the electrode [197]. Recently, cell permeable fluorescent probes have been developed; some of them are based on probe quenching by O2 resulting in a reduction in fluorescence or in O2-dependent red shift of fluorescence [198].

Considering the increasing interest in cell metabolism and the need for an accurate and sensitive method to assess mitochondrial oxidative phosphorylation function, two new systems have been developed that allow monitoring cell respiration in response to external stimuli. These systems are the high-resolution respirometry Oxygraph-2k (O2k, Oroboros Instruments, Austria) and the sensitive high-throughput Seahorse XF Extracellular Flux Analyzer (Seahorse XF, Seahorse Bioscience Inc.). Nowadays, both these methods are widely used for the evaluation of peripheral blood cells bioenergetics, including platelets [135,199,200]. The O2k measures, in real time, the rapid changes in oxygen concentration (nmol/mL) and oxygen consumption (pmol/sec/mL), both markers of OXPHOS, using a polarographic sensors. It allows testing platelets response to a multitude of injectable reagents using small quantities of initial sample. However, it requires constant monitoring and only two samples can be analyzed simultaneously, resulting in a very low throughput [201]. The extracellular flux analyzer (XF) uses a novel fluorescent sensor-containing biocartridge to measure O2 consumption rate (OCR), and extracellular acidification rate (ECAR), associated to mitochondria respiration and glycolysis, respectively [199]. The system is fully automated, and it enables high-capacity sample analysis, since a single plate with multiple samples can be analyzed with high resolution [201]. Mitochondrial bioenergetic profiles can be obtained measuring basal respiration and OCR/ECAR after the injection of sequential injection of inhibitors of OXPHOS. The injection of oligomycin, a specific inhibitor of the ATP synthase, induces a drastic reduction in OCR and it allows determining the rate of oxygen consumption that corresponds to ATP synthesis, while the oligomycin-insensitive rate is considered as proton leak across the inner mitochondrial membrane. FCCP (carbonyl cyanide-4-(trifluoromethoxy)phenylhydrazone), an uncoupler of the ETC, was used to determine the maximal respiration rate. The difference between basal respiration and this FCCP-stimulated OCR represents the reserve capacity of mitochondria, which corresponds to the maximal potential respiratory capacity of cell under stress conditions and/or increased energetic demands. Antimycin A, an inhibitor of complex III, is used to completely inhibit mitochondrial electron transport and to assess non-mitochondrial oxygen consumption.

These advanced methods, coupled with analyses of oxidative stress markers and platelet activation/aggregation assays could be useful for understanding platelet bioenergetic profiles in normal and pathological conditions, but they still lack standardization to find applications in clinical routine.

## 8. Clinical Transferability

As outlined, platelet activation is influenced by the balance between oxidative stress and redox state.

However, the clinical implications of oxidative stress in platelet function and thrombosis are still controversial.

So far, specific disease states that have been linked with platelet oxidative stress include obesity, hypertension, insulin resistance and type II diabetes. Overall, these are all part of the so-called metabolic syndrome. Obese and hypertensive patients typically show activated circulating platelets, increased platelet aggregation and formation of platelet-leukocyte aggregates, enhanced endogenous ROS production and reduced antioxidant status, NOS activity and NO bioavailability [202,203]. In type II diabetic patients, intracellular calcium homeostasis is compromised, probably due to increased hydrogen peroxide and peroxyl radical levels, leading to platelet hyper-reactivity and hyper-aggregability [204]

Given this scenario, the antioxidant activity of natural compounds has been investigated in order to contrast platelet endogenous oxidative stress and associated cardiovascular diseases. These compounds include not only well-known molecules with widely investigated antioxidant properties such as polyphenols and flavonoids—which appear to also be active in platelet-related oxidative stress—but also other novel bioactive plant components.

The protective effects of the polyphenolic and flavonoid-rich extract from berries of Aronia Melanocarpa (containing anthocyanidines, phenolic acids and quercetin glycosides) on platelet oxidative-stress biomarkers have been investigated by Kedzierska et al. [205] and Olas et al. [206]. The first work the analyzed oxidative/nitrative modifications of blood platelet proteins in breast cancer patients, by measuring the level of biomarkers of oxidative/nitrative stress such as carbonyl groups, thiol groups and 3-nitrotyrosine. The authors demonstrated that the polyphenol rich extract of A.Melanocarpa is capable, in vitro, to reduce thiol groups and 3-nitrotyrosine in platelet proteins, therefore counteracting platelet oxidative stress induced by cancer. This compound proved to be active also in the reduction of other platelet oxidative markers, namely the level of 8-epi-prostaglandin F(2) and glutathione amount, together with platelet activation markers, such as GPαIIbβ3 [206].

Based on these findings, Sikora et al. [207] investigated the effects of dietary supplementation with A. Melanocarpa extract on platelet aggregation, clot formation, and lysis in patients with metabolic syndrome. After 1 month, the authors observed an inhibition of platelet aggregation and a beneficial reduction in the overall potential for clot formation and fibrinolysis.

Argan oil produced an antioxidant effect by reducing platelet malondialdehyde levels, and increasing platelet glutathione peroxidase activity [208]. Experiments in rats indicated that treatment of platelets by argan oil (0.2, 0.5, 1, and 2%) prevents platelet aggregation induced in vitro and ex vivo by thrombin or epinephrine [209].

Aqueous extract of medicinal plant Conyza canadensis acts as a free radical scavenger, reducing O2− generation, and inhibiting the oxidation and nitration of proteins in blood platelets treated with peroxynitrite ONOO− [210].

Finally, Cinnamtannin B1, found in Cinnamomum verum, is a type A proanthocyanidin which exerts an effective antioxidant action through the inhibition of endogenous ROS generation in platelets derived from type II diabetic patients [211,212].

Advances in the knowledge of the functional role of ROS in platelet biology and the mechanism of action of plants with antiplatelet effect will hopefully provide new approaches to develop pharmaceutical strategies to promote cardiovascular health.

## 9. Conclusions

Strict relationships between platelet functions and redox state exist. Indeed, platelets are both a source and target of ROS and a fine balance between ROS production and ROS detoxification divide platelet physiology from pathology. Changes in redox status occur during platelet activation, and several platelet activation pathways lead to intraplatelet ROS production, primarily via NOX and mitochondria. In turn, platelet-derived ROS, as well as ROS/RNS, act as second messengers and can influence different signaling pathways, which enhance agonist-induced platelet functions and promote platelet recruitment and aggregation. Therefore, enzymatic and non-enzymatic antioxidant systems have a key role to prevent cytotoxic effects of ROS and to regulate redox-sensitive signaling pathways in platelets.

Platelet redox systems have important clinical implications. Of note, several pathological conditions show both altered platelet function and imbalance of redox homeostasis; both these aspects are often part of a vicious circle that may lead to serious consequences. Platelet redox status looks to be a promising biomarker, as well as a good candidate target for cardiovascular and inflammatory diseases prevention and treatment.

## Figures and Tables

**Table 1:** Fluorescent probes to detect ROS levels in platelets.

Probe	Detected ROS	Maximum Excitation Spectra (nm)	Maximum Emission Spectra (nm)	Limitations and Artefacts	References
CellROX^®^ Green	H_2_O_2_ NO ONOO− O•2-	485	520	Antioxidants	[158]
CellROX^®^ Orange	H_2_O_2_ HO• NO ONOO− O•2-	545	565	Antioxidants	[158,159]
CellROX^®^ Deep Red	O•2- HO•	644	655	Antioxidants	[159]
C11-BODIPY^581/591^ (membrane)	HO• ROO•	488	520	Hemolysis Antioxidants	[159,160]
DAF-FM	NO	488	520		[161,162]
DCFH-DA/ DCF	HO• ROO• •NO ONOO− Indirectly H_2_O_2_	495	529	Hemolysis Self-propagation of DCF radicals Esterase inhibitors Plasma esterase in whole blood or PRP EDTA and citrate Antioxidants	[157,163,164]
DHE/2OH-Et+	O•2-	400	580	Heme enzymes interference Redox-cycling Auto-oxidation	[151,165,166]
MitoSOX^TM^ Red	Mitochindrial O•2-	510	580	Mitochondria overload	[151,167,168]

2OH-Et+: 2-hydroxy-ethidium; C11-BODIPY581/591: 4,4-difluoro-5-(4-phenyl-1,3-butadienyl)-4-bora-3a,4a-diaza-s-indacene-3-undecanoic acid; DAF-FM: 4-amino-5-methylamino-2′,7′-difluorofluorescein diacetate; DCF: 2′,7′-dichlorofluorescein; DCFH-DA: dihydrochlorofluorescein diacetate; DCF: 2′,7′-dichlorofluorescein; DHE: dihydroethidium; EDTA: ethylenediaminetetraacetic acid, H2O2: hydrogen peroxide; HClO: hypochlorous acid; HE: hydroethidine; MDR: multidrug resistance; NO•: nitrogen monoxide; NO•: nitrogen dioxide; O•-: superoxide radical; HO•: hydroxyl radical; ONOO−: peroxynitrite; PRP: platelet-rich plasma; ROO•: peroxyl radicals.

## Abbreviations

•NO	nitric oxide
•NO_2_	nitrogen dioxide radical
•OH	hydroxyl radical
∆Ψm	mitochondrial membrane potential
2OH-Et+	2-hydroxy-ethidium
3-NO-Tyr	3-nitro-tyrosine
8-iso-PGF2α	8-iso-prostaglandin F2α
αIIbβ3	glycoprotein GPIIb/IIIa
AA	arachidonic acid
ADP	adenosine diphosphate
ADAM	disintegrin and metalloproteinase
AMI	acute myocardial infarction
BHT	butyl hydroxytoluene
C11-BODIPY581/591	4,4-difluoro-5-(4-phenyl-1,3-butadienyl)-4-bora-3a, 4a-diaza-s-indacene-3-undecanoic acid
CAA	cerebrovascular amyloid angiopathy
CaM	calmodulin
CAT	catalase
CD40L	CD40 ligand
CD62p	P-selectin
COX	cyclooxygenase
DAF-FM	4-amino-5-methylamino-2′,7′-difluorofluorescein diacetate
DCF	2′,7′-dichlorofluorescein
DCFH-DA	dihydrochlorofluorescein diacetate
DHE	dihydroethidium
DNDPH	2,4-dinitrophenylhydrazine
DNP	dinitrophenyl
DPI	diphenyleneiodonium cloride
DTNB	5,5′-dithio-bis (2 nitrobenzoic acid)
ECAR	extracellualr acidification rate
EDTA	ethylenediaminetetraacetic acid
ELISA	enzyme-linked immunosorbent assay
eNOSIII	endothelial-like NO synthase III
EPR	electron paramagnetic resonance
ETC	electron transport chain
FAD	flavin adenine dinucleotide
FCCP	carbonyl cyanide-4-(trifluoromethoxy)phenylhydrazone
FCM	flow cytometry
FcRγ	Fc receptor γ-chain
GC-MS	gas chromatography coupled with mass spectroscopy
GPIbα	glycoprotein Ibα
GPVI	glycoprotein VI
GPx	glutathione peroxidase
GSH	glutathione
GSSG	glutathione disulfide
GST	glutathione S-transferase
H_2_O_2_	hydrogen peroxide
HClO	hypochlorous acid
HE	hydroethidine
Hic-5	focal adeshion adapter protein
HNE	4-hydroxy-2-nonenal
HO-1	heme oxygenase 1
HPLC	high-performance liquid chromatography
IP3	inositol 1,4,5-trisphosphate
ITAM	immunoreceptor tyrosine-based activation motif
LC-MS	liquid chromatography mass spectrometry
MAPKp38	P38 mitogen-activated protein kinases
MDA	malondialdehyde
MDR	multidrug resistance
MKs	megakaryocytes
mPTP	mitochondrial permeability transition pore
NAC	N-acetylcysteine
NAD(P)H	nicotinamide adenine dinucleotide (phosphate)
NO-GC	NO-sensitive guanylyl cyclase
NOS	nitric oxide synthase
Nrf2-Keap1	nuclear factor eruthroid-2 related factor 2/kelch-like ECH-associated protein
O•2-	superoxide anion
O_2_	molecular oxygen
O2K	Oxygraph-2k
OCR	O_2_ consumption rate
OH−	hydroxyl ion
ONOO-	peroxynitrite
Ox-LDL	oxidized low-density lipoprotein
OXPHOS	oxidative phosphorylation
P2Y1 and P2Y12	purinergic G protein-coupled receptors
PAR1 and PAR4	protease activated receptors
PDIs	platelet disulfide isomerase
PGH_2_	prostaglandin H2
PI(3,4)P	phosphatidylinositol 3,4-biphosphate
PI3K	phosphatidylinositol 3-kinase
PKC	protein kinase C
PLA2	phospholipase A2
PLCγ2	phospholipase Cγ2
PLTs	platelets
PRP	platelet-rich plasma;
PrxII	peroxiredoxin II
PS	phosphatidylserine
Pyk2	proline rich tyrosine kinase 2
RNS	reactive nitrogen species
ROO•	peroxyl radicals
ROS	Reactive oxygen species
sCD40L	Soluble CD40 ligand
SH3	Src homology-3
SHP-2	Src homology region 2-containing protein tyrosine phosphatases 2
SOD1/2	superoxide dismutase 1/2
Syk	spleen tyrosine kinase
TBA	thiobarbituric acid
TNB	5-thio-2-nitrobenzoic acid
TP	thromboxane receptor
TRAF4	tumor nescrosis factor associated factor 4
Trx	thioredoxin
TXA_2_	thromboxane A_2_
U46619	thromboxane A2 analog
VWR	von Willebrand factor
X-linked CGD	X-linked chronic granulomatous disease
XF	extracellular flux analyzer
XO	xanthine oxidase
